# Applying implementation science to infectious disease emergency preparedness and response

**DOI:** 10.3389/fpubh.2025.1622618

**Published:** 2025-07-16

**Authors:** John Øvretveit

**Affiliations:** LIME/MMC, Karolinska Institutet Medical University, Stockholm, Sweden

**Keywords:** implementation, emergency, infectious disease, crisis response, disaster management, adaptive implementation

## Abstract

Some infectious disease outbreaks present an emergency and a potential disaster scenario. Examples include a new virus similar to or more infectious and deadly than COVID-19 or Ebola, such as the 2025 MERS-like virus. Although good practices for general emergency preparedness and management can be used to plan, there are different and less-known preparations and responses needed for some infectious disease outbreaks and continuing and evolving crises. The objectives of this article are to provide guidance about how to plan and respond to these types of emergencies more effectively by using implementation science knowledge as well as lessons from COVID-19 and other infectious disease emergencies. This narrative review gives guidance for healthcare service delivery leaders at different levels of a healthcare system and easy to understand and use resources for leaders to improve their infectious disease emergency preparedness and operational actions in a disaster scenario. The implementation science guidance covers: interventions, adaptation to context, iteration, coordination for alignment, facilitation, and how to use behavior and organization change models and theories. It also provides researchers with an overview of issues and frameworks to help focus their research designs and data collection to study intervention implementation processes and outcomes for infectious disease outbreaks.

## 1 Introduction

Leaders and researchers are already overburdened and directed to spend their efforts on meeting many “priorities” and requirements. Preparations to respond to and plan for emergency infectious disease outbreaks (IDO) is work that can be easy to put to one side. Researchers facing funding cuts and more narrow funding calls find it difficult to study emergency IDOS, and often do not provide specific guidance to leaders. “Why spend time on something that may never happen, even if the consequence of the event would have a high impact? We already have general disaster response guidance and plans that we will use.”

Were there delays in changing behavior and organization during the COVID-19 outbreak? How quickly could emergency departments and primary care units reliably practice the new guidance as we gained knowledge about the virus? Could leaders have been more effective and faster in achieving these changes? This article shows how leaders can do so, and researchers can help them, by using implementation science (IS) knowledge. Implementation science is, “the systematic study of how to design and evaluate a set of activities to facilitate successful uptake of an evidence-based health intervention. ‘Evidence-based’ refers to interventions that have undergone sufficient scientific evaluation to be considered effective and/or are recommended by respected public health or professional organizations”.

It is certain there will be infectious disease outbreaks, but it is uncertain when or whether they will present an emergency or a potential disaster scenario, such as the 2025 MERS-like virus (1,40). The relaxation of and low uptake of preventative measures increases this likelihood, as does less available and timely data for researchers and leaders (1). As a joint researcher-practitioner team in Stockholm our experience was that general disaster response guidance and protocols were of limited use in the COVID-19 outbreak and for Ebola precautions (2). This was also discovered also during large scale urban wildfire emergencies such as the Los Angeles 2025 fires. The emergency evolved into different stages needing different response and implementation strategies: containing the fire and immediate health issues was different to recovery, rebuilding and the type of community and trauma support needed, for example with new evidence of harmful air quality and water contamination (3).

The purpose is to provide in one article guidance based on best available implementation science evidence to enable leaders and researchers to better respond to the next IDO. It is based on one implementation science researcher’s review of the evidence and experience working for Stockholm healthcare, including the emergent and continuing emergency of the 3 years of the COVID-19 IDO. Our experience and earlier review identified gaps in knowledge that this narrative review seeks to address by providing concepts and resources for researchers and managers or implementation leaders. These gaps include a lack of knowledge about how to respond flexibly and continually to an evolving crisis after the initial impact of the emergency; also, a lack of clear concepts about the different aspects of implementing responses to an emergency and the evolving crisis; and there is no simple research informed guidance for managers, implementers and researchers unfamiliar with implementation science.

The article starts with the COVID-19 emergency as an example, highlighting the elements from implementation science that were important in helping and hindering the Stockholm response. The third section then elaborates on this knowledge, giving the sources where more details can be found, as well as the limitations of this overview. The fourth section provides recommendations for quick reference for leaders and researchers to turn to in the next emergency and a potential disaster scenario, highlighting how important it is to modify these for their context and the uncertainties.

### 1.1 What this adds

- Knowledge about continuing evolving crises rather than discrete short duration emergencies,
- Evidence based practical guidance for leaders at all levels of a healthcare system,
- Concepts and models to help researchers to study infectious disease outbreaks and provide actionable and rapid knowledge to implementers,
- Practical illustrations from an exaple of 3 years of evolving response implementation and research.

### 1.2 Terms used in this article

Infectious disease emergency: a sudden incidence of an infectious disease posing actual or likely serious harm to humans, because of severity of impact and/or speed of spread, and that calls for rapid action (4).

Intervention content: the new better way of working (or for the carer or patient - the new better behavior or lifestyle) (5).

Implementation strategy: actions taken to enable providers, patients or carers to perform the new better way (5).

Context: anything that is not the intervention. The environment within which the new better way is intended to be taken up by providers, patients, carers or organizations (5). Only some features influence implementation.

Implementation research: the activity undertaken to describe, understand, explain and evaluate how healthcare “takes up” a proven “improvement-change” in one unit, service or in many. Implementation research may or may not evaluate ultimate outcomes for patients, but usually evaluates how much the “improvement-change” is “taken up” by a service (5).

## 2 Example

This summary of the COVID 19 emergency and evolving crisis in Stockholm highlights the elements from implementation science that were important in helping and hindering our response and for the rapid impact research that a joint researcher-practitioner team undertook. The example refers to the initial emergency in phase 1 between March 2020 and October; Phase 2 was November 2020 to May 2021 preparation for and providing vaccines; Phase 3 June 2021 to June 2022 responding to new variants and providing booster doses, Phase 4 June 2022 to December 2023 recovering and dealing with staff shortages. The dates are important to show how this epidemic was, for us in Stockholm, best described as an emergent- and continuing- emergency, rather than a time limited event. Unique features of the context were that our schools never closed and there was no formal lockdown but, like other countries, there was the tragedy of avoidable deaths among nursing home residents did occur (6).

In phase 1 Our first emergency department visit by a patient with confirmed COVID-19 was in early March 2020, after the top health system management had implemented an emergency management system and structure (EMS) at the end of February (2). We consided the EMS to be a management intervention and the implementation was through direction and feedback on effectiveness. In the early days there was little knowledge about virus transmission, but then came more evidence, and then vaccination and new variants, all of which changed the situation and called for different responses with different implementation activities and pace. Burnout in all phases caused a vicious cycle with short staffing increasing the burden for others, and growing misinformation and mistrust of experts, and then budget cuts, all creating new crises, calling for continuing leader and researcher responses.

The intervention EMS NATO model, not used before in Stockholm, involved leaders at all levels. Effective implementation took time because leaders had to learn their new role and their contribution to the decision-making process. This also included changing the daily meetings procedures to shorten discussions about actions by creating sub-groups to gather information and make recommendations for later meetings. Delays by higher-level leader groups to approve actions or make other decisions led to lower-level leaders, such as heads of emergency departments, breaking usual protocols when necessary to protect staff and patients. This informally modified the emergency management structure to be more fit for purpose. During the 2020 summer, progress was made in implementing data systems to provide leaders with more timely and reliable information, such as available service capacity and infection rates. By Phase 2 in the winter of the first year of the pandemic, preparations had to be made quickly to procure vaccines and implement mass vaccination clinics at certain sites.

In phase 3 during the second year, more infectious virus variants and better evidence about transmission called for implementing new responses and influencing behavior change. In phases 2 and 3, new information about disproportionate COVID-19 morbidity and mortality and vaccine uptake among non-EU residents led to the implementation of peer community health workers visiting residences and vaccination buses. By the third year, a crisis continued in areas of service staffing, burnout, the increasing impact of long COVID, as well as budget cuts, all calling for new and different responses to be implemented.

## 3 How IS can help leaders and researchers with emergency IDOs

The following shows how implementation science helped a more effective response in the above example, and also how IS can help leaders and researchers with other emergency IDOs. These elements are later summarized in guidance in the section after, with recommended IS tools.

### 3.1 Interventions

The first contribution of IS is the need to define the interventions (or innovations) to be implemented at different times in response to the emergency, and to differentiate this from the implementation actions. The intervention is the intended new, “better way of working” or lifestyle change for the emergency situation at the time. Examples are different infection control interventions and changes as we gained knowledge about virus transmission. In the COVID-19 example, the first intervention was, on the last day of February 2020, a NATO emergency management model directed by the top management team. Leaders at each level thus had to change their behaviors to adjust to their new roles and include this work with their regular roles. The intervention was the NATO model, and the implementation was the behavioral change of the leaders to effectuate the new management structure. Another intervention was procuring personnel protective equipment (PPE) and directing PPE use.

Implementation science has found that distinguishing and describing the intervention from the implementation actions is essential to plan and carry out the implementation actions (5, 7, 8). Also, is defining the actions to be carried out by staff at each level to use PPE effectively. Implementation is the actions we observe, and the effective use of PPE is the outcome of the intervention and the implementation (5). Another example is communication as an implementation activity and the content of the communication as the intervention. To implement vaccine uptake in certain ethnic groups we facilitated Imans and other faith-based leaders to communicate facts and encouragement about vaccines. More practical guidance about this implementation strategy is given in FEMA (9), Santibañez et al. (10).

### 3.2 Adaptation to context

The second contribution of IS to a more effective emergency response is understanding how to adapt appropriately both the intervention and the implementation actions. For PPE, some implementation actions were not carried out by higher level leaders and consequently many heads of services did not have any PPE and could not do their implementation work of training and supervision. Some heads of service and staff improvised to implement the intervention by buying PPE, making their own, and wearing adapted trash bags. Implementation science highlights a tension that leaders, staff and others have to resolve: between copying the intervention exactly (the correct PPE) and adapting the intervention if necessary (11). Some personnel were able to get masks, some were not, so they copied the principle of mouth and nose covering with what was available. Later, as the context changed with more evidence about effective masking and recommended PPE available, leaders changed their implementation actions to give new communications, training and supervision and staff and citizens changed their behaviors, for example to ensure proper fit of recommended masks.

### 3.3 Iteration

Leaders often use review meetings to decide whether to change their actions and staff practices. IS has found structured iteration to be more effective for more complex changes to maximize the use of available, reliable information for adaptation to context. Structured iteration borrows proven tools from quality improvement, such as the Plan-Do-Study-Act (PDSA) method to carry out small changes and to use data to find if the change is effective, as well as process mapping and pathway guidance (12–15). In the Stockholm example, the research-practitioner team provided the mass vaccination operational leaders with their process analysis of the mass vaccination clinic to help identify bottleneck-delays and carry out fast PDSA test of change cycles (15–17). In emergency situations it sometimes more effective to carry out a PDSA to test a staff suggestion to find out if it is likely to work and give staff the evidence and motivation to carry out the change in their workplace and on a larger scale.

### 3.4 Coordination for alignment

In many emergency situations, different services in different sectors respond, but what each does impacts other services. General disaster management practice is to establish coordination between services to reduce negative consequences and optimize the overall impact of services on individual or population well-being (17, 18). This may be by a planned EMS coordination structure and/or an *ad hoc* structure for the emergency. IS evidence shows that aligning the actions that leaders take so that the actions contribute to the same objective is more effective, especially in a changing situation (19). Examples are aligning actions by primary care with those of hospital outpatient, emergency and inpatient actions to ensure a smooth transition or parallel care of patients between needed services (horizontal coordination.) This requires a coordination structure, including information technology systems, designated coordination roles as well as regular meetings between the relevant parties to understand and adjust how their actions affect each other. Agreed patient pathways between services are also a coordination structure that can ensure the sum total of the actions is greater than the parts and the right patients get to the right persons to help them at the right time (20). Supplementary and different structures were also needed including inter-sector coordination between the county (healthcare) and municipalities [social care (19, 21)]. Some solutions are a Network structure for collaboration and coordination and targeted communication (22).

Similarly, IS has found that aligning the actions of leaders at different levels (vertical coordination) increases the effectiveness of the actions. Higher level leaders create an enabling context for lower level leaders, such as by delegating increased authority to purchase items locally, for example protective preventative equipment (PPE). In the example, the planned formal EMS did not enable the right changes or fast enough changes to be made by higher levels in the COVID-19 emergency. A theory useful for implementation planning and operational response helped us to explain how fast, locally tailored responses made by service delivery units did not have negative impacts on other services and cause system sub-optimization. This was a theory of decentralization, combining the concepts of delegated authority, management capability, and accountability for appropriate management decentralization (23).

### 3.5 Facilitation

IS has found that implementation facilitation is effective to help leaders to carry out faster and more wide-scale change to implement interventions (24). However, in most IS research the researchers or other resourced and trained faciliators provide this function, and many organisations do not have this resource available, or a quality improvement function that can sometimes provide facilitation. In Stockholm, the research-practitioner team was able to support the management team and other crisis projects with independent general meeting facilitation and also with reminders about behavioral and organizational change tools that could help speed the changes. Implementation research giving more details includes practical guidance for implementation facilitators (8, 25, 26) and studies with details of facilitation (24, 27–29).

### 3.6 Codesign implementation

Designing the implementation actions together with those who will take up the intervention helps to make the actions acceptable and more likely to be performed. It also prepares and motivates those involved to take up the intervention. IS studies report that guidance on the role people can take to support implementation is frequently overlooked and even those involved in co-design are not included in the implementation process, for example to help design and make adjustments (11). IS has found co-design to be an effective part of implementation. However, it take time and skills to perform effectively and many researchers or implementers do not have this. Also the right stakeholder are needed as well as trust that their time will be used and ideas folowed through and this does not always happen. Examples of implementation codesign are involving both providers and patients in planning the implementation of a design for a long covid clinic or a mass infectious disease testing facility. Different approaches to implementation codesign are described in Lynch et al. (30), Walker et al. (31), Harrison et al. (32), Kehoe et al. (33), and for resource constrained settings in Singh et al. (34).

### 3.7 Peer implementation

A powerful way to enable behavior change is research informed peer meetings and support. Peers can be leaders of equal status, who teach and facilitate their peers. For example, about how to change practice to use a new intervention, or respected doctors teaching and facilitating effective communication practices to help implement an intervention. However, to be effective peer implementation usually needs trained peer leaders who know how to enable meetings, and an organisation to arrange and manage meetings or a virtual platform if this is used. Most research on the conditions for effective peer implementation has been carried out for peer support for mental health and addiction challenges (35).

### 3.8 Behavior and organization change models and theories

IS provides researchers with several models to help plan the design and data collection in a study Nilsen (36). Some also have been helpful for leaders and practitioners to enable more effective and sustained change by practitioners or patients. One used in the Stockholm example was the capability–opportunity–motivation–behavior (COM-B) behavior change model (37, 38). COM-B is a guide that shows how some features of the context combines with motivation and opportunity to influence the behavior change that is needed in leaders, practitioners and patients. In the Stockholm example, practitioners needed to change their behavior to follow the infection protection intervention. Most who trusted the instructions were motivated to change their behavior to protect themselves and others. They also had the personal capability to make the behavior changes: they had the physical capability to put on and remove the PPE and mental capability to remember the sequence for doing this. What was missing was the opportunity: the availability of PPE, and for some in emergency rooms, the time to put on and remove the PPE. Sometimes, when the opportunity was available, the culture and social norms worked against the opportunity for example by influential leader not using PPE appropriately. This and other behavior change models that are useful to guide the implementation of different IDO interventions are described in West et al. (39).

Resilience is not an emergency response but a capability that is demonstrated in the way an organization or individual copes with a crisis. Implementation science uses resilience theory to help leaders to plan and also to respond to a continuing crisis so that future events in the crisis and other emergencies are better responded to, rather than further degrading the response capability. Resilience theories are also useful to researchers to help understand emergency responses because they predict how the organization will respond (40). An example of the use of resilience theory for both these purposes in the Stockholm crisis was the development and implementation of elements of resilience as part of one hospital’s response to COVID-19. The study describes how the organization was able to perform and also develop its anticipatory capabilities and identify critical events, and then develop coping responses, and adapt to make this part of new activities (41) (Table 1).

**Table 1:** How implementation science can help leaders and researchers with emergency infectious disease outbreaks.

Implementation science is useful to	Definition	Example
Define the intervention and the implementation actions	The intervention is the new “better way” of doing something, that then has to be put into practice. The implementation actions are what people do to put the intervention into practice.	A vaccine is an intervention. Implementation actions include delivering the vaccine to the clinician, enabling the clinician to give the vaccine correctly and providing information. A team based care model is an intervention that will need implementation actions to change practice and organisation to put it into practice
Adapt the intervention and/or the implementation actions to the context	Adaption is appropriately adjusting the intervention and/or the implementation actions to fit the context, without loosing its effectiveness.	Adapting a vaccine to be effective for a new virus strain. Adapting the implementation actions to be suitable for a specific population group such as culturally sensitive translation of information and training of clinicians.
Iteration of the intervention or the implementation actions to improve it or adjust to a new context	Cycles of revising, checking, and keeping or discarding the changes.	For an intervention such as a temporary vaccine clinic, implementation cycles to check for delays or long waiting, then trying a change to the clinic flow, and checking the change and then keeping or discarding the change.
Alignment to establish coordination between services, sectors, and/or levels.	Aligning the change actions of different service, sectors and/or levels of organisation to optimize the overall impact of services on individual or population well-being	To enable the smooth transfer of patients out of hospital to home agree with primary and home care the information to be provided and the support needed from hospital specialists, for individual patients or groups of patients.
Facilitation	Making something possible or easier, or the act of helping others to reach a solution.	A facilitator or manager with facilitation leadership skills guides a group through steps to design and implement a team based care process. The facilitator does not need to be an expert in the intervention, so long as someone who is an expert is involved.
Codesign implementation	Designing the implementation actions together with those who will take up the intervention.	Implementers involve people in deciding the best actions to enable other people from their population take up a vaccination. Multiple stakeholders may also be involved in codesign, for example in planning a temporary vaccination clinic.
Peer implementation	People with experience in the intervention lead, or facilitate, their peers to take up the intervention in practice and share their experience.	Clinicians meet to share experience and evidence about the best way to answer patients questions or hesitation about a vaccine. People experiencing substance abuse challenges meet to share ther experience of effective ways to manage their challenges. Peer implementation usually needs a trained and trusted facilitor.
Behavior and organization change models and theories	Ideas and frameworks that help to guide data collection or actions to implement an intervention	The COM-B guide to show how some features of the Context combines with Motivation and Opportunity to influence the Behavior change that is needed in leaders, practitioners and patients to take up the intervention. A logic model framework to guide design or data collection by separating and filling in the inputs, activities, implementation outcomes and context descriptions.

## 4 Discussion and recommendations

This overview and the guidance is limited in a number of ways, and readers should be cautious and discriminating in applying the lessons to their situation. In low resource settings with limited digital technology for communication and data collection and analysis, it is more challenging to use some of the implementation methods for coordination and iteration. In these situations, regular local face to face meetings, informed with experiential evidence are more needed, but are also difficult because of concerns about infection spread at the meeting and travel challenges. If feasible and there is internet connection, smart phones for video meetings are possible and useful to involve distant rural staff. Decision tools and guides for resource constrained settings are described in Singh et al. (34), Harrison et al. (32), Nilsen (36), Kehoe et al. (33), and Woodward et al. (42).

Another limitation is the article focus on longer term and evolving IDOs and some of the guidance may be less relevant to short-term emergencies. This overview also assumes the intervention is proven to be effective and concentrates on what are effective actions to enable take up of the intervention. During the COVID-19 epidemic we saw that some interventions were not effective and even harmful, so implementing them effectively caused more harm. It noted how to evaluate implementation, not how to evaluate the intervention, although some IS research designs allow both (43).

For researchers, especially those less familiar with IS, the overview can be used:

- Before data gathering, for example, building on the implementation features that previous research suggests are important for appropriate, effective and fast implementation to respond to a changing situation. This can help deductive observational studies, or hypothesis testing for experimental implementation studies,
- After data collection, to interpret, understand or explain the documented implementation actions at different times that are made to respond to the evolving situation.

Experiential evidence is often be biased, but it may give an indication of what helped and hindered the response in the leader’s setting. Some experience with effective implementation actions for responding to influenza outbreaks was helpful for leaders, and some was not. Leaders and researchers will need to choose which factors are most relevant to their infectious disease outbreak and situation. The following, and Table 2 after, summarizes evidence-based implementation guidance for leaders and researchers considered in the earlier section with a focus on practical resources that they can quickly use.

**Table 2:** Recommendations, depending on the intervention and the context.

Recommendation	Comment	Reference and resources
1. Define what is the intervention and what are the implementation actions.	To do this it is useful to recognize the following types of intervention or innovation, which will need different implementation actions: Those directed at patients or clinicians, such as a intervention for lifestyle changes, with implementation actions that include training, providing information and motivation. Those directed at small organisational units such as a primary care health center,with implementation actions that include faciliation, training, and checklist tools. Those directed at large organisations with many organisational units such as a hospital, with implementation actions to enable small unit leaders and higher level leaders to take up the organisational intervention or innovation. Those directed at large health systems with many large organisations, with implementation actions to enable leaders at different levels to take up the organisational intervention or innovation.	Practical resources are given in Implementation Hub (44) and AIRN (45).
2. Adapt appropriately the intervention and/or the implementation actions to the context	Interventions often do not survive contact with different practice settings and healthcare organisations. Even settings similar to the setting within which the intervention was proven will throw up barriers to implementation. Usually it is necessary to modify the intervention to enable uptake. The danger is modifying it so much that the principles that made it work eleswhere are not well adapted by implementers to the new setting. Resolutions are to get data to check implementation progress, or use a adaption tool suited to the intervention and setting, as described in the references.	Practical resources are given in McCormack et al. (46) and Moore et al. (47).
3. Continually revise/iterate the intervention or the implementation actions to respond to the changing context	To help adaption, regular structured iteration is necessary to respond to the changing context and improve the outcomes, using reliable data about implementation progress.	Plan-Do-Study-Act (PDSA) small scale test of change a the mass vaccination clinic to adjust the pathway through the clinic in response to changing demand for vaccinations (53). Resources for more details are given in the Getting to Outcomes and Interactive Systems Framework implementation tool (48, 49)
4. Coordinate continually to ensure alignment	It is difficult to make time for meetings for cross-service and cross-sector built into structures for coordination, and to management meetings for different level leaders. But it is necessary to do so to enable implementation and avoid sub-optimal system effects. For extended emergency responses, effective coordination is challenging, and even more necessary because different services and sectors will be adapting their implementation or the same intervention to their changing contexts.	Practical details and resources to carry this out and to study it are provided in the CASPI toolkit (53, 50, 51, 54, 2).
5. Use facilitators to help design and carry out implementation strategies more effectively.	The facilitator, who may be an organisational leader and may be trained in management facilitation, does not need to know the details of the intervention, but needs to involve in implementation meetings someone who does.	Practical guidance is given in Ovretevit and Tortolani (53), QUERI (26), and Olmos-Ocha et al. (8).
6. Codesign implementation	If there is time, then co-design carried out well can speed and ease implementation can. A necessity is to include the “right people” who will take up the intervention. Co-design for research is sometimes required by funders, for example patient involvement in desigining the research.	Codesign for resource constrained settings is described in Sing et al. (34), Harrison et al. (32), Kehoe et al. (33), and Woodward (42). Practical guidance for researchers is given in NSW (52).
7. Use peer implementation	Peer implementation can be effective if meetings of peers are led well. Peer implementation in continuing crises can help changes and adaptations, for with peer groups for professionals, other staff, patients and communities.	Practical guidance is given in SAMSHA (35).
8. Use behavior and organization change models and theories as appropriate	There are many frameworks and models, so it is possible to choose one suited to the implementation setting for the purposes of the project	A general theory to guide implementation and a practical guide to use it is the COM-B model given in Michie et al. (37) and Social Change (38). For researchers, models to help plan the design and data collection are given in Nilsen (36).

### 4.1 Define what is the intervention and what are the implementation actions

An example of an intervention is providing and directing the use of PPE. Implementation actions are what is done to enable people to obtain and use PPE appropriately, such as procuring, distributing, training and supervision. A vaccine is an intervention, and the mRNA vaccine was an innovation as well. Implementation actions are what is done to enable providers and the public to obtain and take up the vaccine as appropriate. Practical resources to enable this differentiation between intervention and implementation with more details are given in The Active Implementation Hub (44), AIRN (45).

### 4.2 Adaptation to context

An example is how the NATO emergency organization structure was adapted to healthcare and the IDO in the Stockholm situation. This was implemented through an interesting combination of informal adaption by lower-level leaders, who decided to act because of delays in decisions by the higher levels, and then a formal adjustment by them to legitimize certain lower-level decisions (2).

Another was improvising PPE mouth and nose covering with what was available when there were no recommended masks and using plastic dustbin bags as aprons. The challenge is to decide how to, and how much to adapt the intervention or the recommended implementation actions from the proven intervention or implementation actions. It may be the adaption loses the “active ingredient” that made the intervention or implementation actions effective. An example was adapting mouth and nose covering by using a scarf, which research showed did not prevent infection. Resources for more details are given in McCormack et al. (46) and Moore et al. (47).

### 4.3 Continually revise/iterate the way you implement the intervention to respond to the changing context

To improve effectiveness of either the intervention or the implementation actions in the situation and ensure appropriate adaption to the changing context, regular structured iteration is necessary. Staff suggestions for changes may need a small systematic test of the change before making it daily practice. An example was using a Plan-Do-Study-Act (PDSA) small scale test of change in the mass vaccination clinic to adjust the pathway through the clinic in response to changing demand for vaccinations (17). Resources for more details are given in the Getting to Outcomes and Interactive Systems Framework implementation tool (48) and in Langly et al. (49).

### 4.4 Coordinate continually to ensure alignment

Give time and effort to meetings for cross-service and cross-sector structures for coordination. Make changes to these and other structures, such information technology systems, if they are not ensuring effective coordination. The same applies to vertical coordination between leaders at different levels to ensure higher levels create the right context for lower-level leaders. In Stockholm the structure for decentralization in primary care was effective in enabling coordination within this service, but the new NATO emergency management structure was not (2).

For extended emergency responses, effective coordination is challenging. But it is even more necessary because different services and sectors will be adapting their implementation or the same intervention to their contexts. If mutual adjustments are not made, then one adaptation may work against the actions being made by others and the synergistic effect on population or patient health will be lost. Practical details and resources to carry this out and to study it are provided in the CASPI toolkit (S4A 2023) and in CIR (50), Joint Commission (51), Ulibarri et al. (22), and Ohrling et al. (2).

### 4.5 Use facilitators to help design and carry out implementation strategies more effectively

Some services have quality specialists skilled in facilitation. They do not necessarily have to know the details of the intervention, just how to help behavioral change and project group processes. Practical guidance is given in Ovretveit and Tortolani (25), QUERI (26), and Ochoa et al. (36).

### 4.6 Codesign implementation

Find and include the right people who will take up the intervention when planning and adjusting the implementation actions. It will help to make implementation faster and more effective and prepares and motivates people to take up the intervention. Implementation codesign for resource constrained settings is described in Singh et al. (34), Harrison (32), Kehoe et al. (33). Co-design for research is sometimes required by funders, and practical guidance for researchers is given, including how to involve the “right people” in NSW (52).

### 4.7 Use peer implementation

Find and train respected peers to guide and motivate people to take up the intervention and follow the implementation actions. Use peer implementation in continuing crises to help with new changes and adaptations, for professionals, other staff and patients. Practical guidance is given in SAMSHA (35).

### 4.8 Use behavior and organization change models and theories as appropriate

Many of the recommendations above are based on evidence and theories from implementation. Use the capability–opportunity–motivation–behavior (COM-B) behavior change model which is the simplest and easy to follow general theory to guide implementation and a practical guide to use it is given in Michie et al. (37) and Social Change (38). For researchers, models to help plan the design and data collection are given in Nilsen (36).

## 5 Conclusion

Most organisations have plans and or guidance for emergencies or disasters, but these are often not well developed for longer term and evolving crises, such as the COVID-19 2–3 year crisis, or provide actionable research informed guidance for leaders, implementers or researchers. Implementation science is often unfamiliar to those working or studing emergencies or disasters, but is a valuable resource especially for extended and evolving crises. The evidence from IS research provides effective and tested implementation concepts models and tools for these crises.

This narrative review drew on this research and implementation experience to provide on overview of the science for planning, responding and studying evolving crises. It summarized practical recommendations based on IS research for leaders, implementers and researchers and gave resources for tools and more details covering how to define the intervention and implementation actions, adaptation to context, iteration, coordination for alignment, facilitation, peer implementation and how to use behavior and organization change models and theories. Future research is needed to give better research informed guidance about appropriate adaptations of different types of interventions in emergencies or disasters to different contexts and especially for low resource settings and services worldwide.
